# Characterization of the complete mitochondrial genome of *Morchella eohespera*

**DOI:** 10.1080/23802359.2020.1797559

**Published:** 2020-07-29

**Authors:** Wei Sa, Qian-Han Shang, Ting Yang, Qi-Yuan Gao, Mi-Li Liu, Jian Liang, Zhong-Hu Li

**Affiliations:** a State Key Laboratory of Plateau Ecology and Agriculture, College of Ecological and Environmental Engineering, Qinghai University, Xining, PR China; b Key Laboratory of Resources Biology and Biotechnology in Western China, Ministry of Education, College of Life Sciences, Northwest University, Xi’an, PR China

**Keywords:** *Morchella eohespera*, mitochondrial genome, phylogenetic relationship

## Abstract

*Morchella eohespera* Beug, Voitk & O’Donnell is a typical black morel species. In this study, using the Nanopore sequencing platform, we characterized its whole mitochondrial (mt) genome sequence. Mt genome of *M*. *eohespera* is composed of circular DNA molecules of 243,963 bp, which encoded 102 protein-coding genes (PCGs), two ribosomal *RNA* genes (rRNA), and 31 transfer *RNA* (*tRNA*) genes. The base composition of *M*. *eohespera* mitogenome is as follows: A (30.40%), T (29.30%), G (20.8%), and C (19.5%). The phylogenetic analysis suggested that *M*. *eohespera* was closely related to the congeneric *M*. *importuna*.

*Morchella eohespera* Beug, Voitk, & O’Donnell (*Morchellaceae*) is a typical black morel species, which widely distributed in the central Europe, North American, and the central parts of China (Du et al. 2016; Voitk et al. 2016). The modern medical research showed that the morel species possesses various physiologically active properties, such as anti-oxidative, anti-inflammatory, anti-microbial, immunostimulatory, anti-tumor, etc. (Nitha et al. 2007). However, in recent years, the overexploitation and climatic oscillations have resulted in the rapidly decline of the natural resources of morel. Therefore, it is urgent to protect and manage the filed resources of morel species. The genome of species provides the important information to the management and conservation of bio-resources. In the study presented here, we characterized the complete mitochondrial genome of *M*. *eohespera*.

The *M. eohespera* M200 strain was collected from Maco River Forest Farm of Qinghai province, China (N:32.6738; E:101.0773). The tissue materials and voucher specimen (No. MCRFF2019200) of *M. eohespera* were stored in Northwest University. Total DNA was extracted from the myclia using the fungal DNA kit (Sangon Biotech, Shanghhai, China) according to the manufacturer’s instructions. Four nuclear gene fragments (*ITS*, *EF1*-*α*, *RPB1*, and *RPB2*) were amplified to species identification. Mitochondrial genome sequencing was performed using Nanopore platform (GOOLA GENE, Wuhan, China). The long reads were assembled by CANU software (Koren et al. 2017). The mitochondrial ORFs were identified automatically with Mfannot and RNAweasel using genetic code 4 (http://megasun.bch.umontreal.ca/cgi-bin/mfannot/mfannotInterface.pl). Functional assignments were performed based on sequence similarity to characterize fungal mitochondrial proteins using BLASTP searches against Nr and InterPro databases. The tRNAscan-SE version 2.0 (Lowe and Eddy 1997) was used to predict *tRNA* genes.

The mitogenome of *M*. *eohespera* was assembled as circular DNA molecules with 243,963 bp in length, which encoded 102 protein-coding genes (PCGs), 31 *tRNA* genes, and two ribosomal *RNA* genes. The overall base composition of *M*. *eohespera* is as follows: A (30.40%), T (29.30%), G (20.8%), and C (19.5%). The complete and annotated mitogenome genome of *M*. *eohespera* has been submitted to GenBank with the accession number MT635617.

The whole mt genomes of *M*. *importuna* and sixteen other fungal species were downloaded from NCBI. The fourteen conserved protein genes (*atp6*, *atp8*, *atp9*, *cob*, *cox1*, *cox2*, *cox3*, *nad1*, *nad2*, *nad3*, *nad4*, *nad4L*, *nad5*, and *nad6*) were used for phylogenetic analysis, the multiple sequence alignment was performed by MAFFT version 7.158b software (Katoh et al. 2002). The phylogenetic tree was constructed *via* RAxML software (Stamatakis 2014) with 1000 bootstrap replicates. The results indicated that *M. eohespera* was closely related to the congeneric *M*. *importuna* with high bootstrap support (Figure 1).

**Figure 1.**
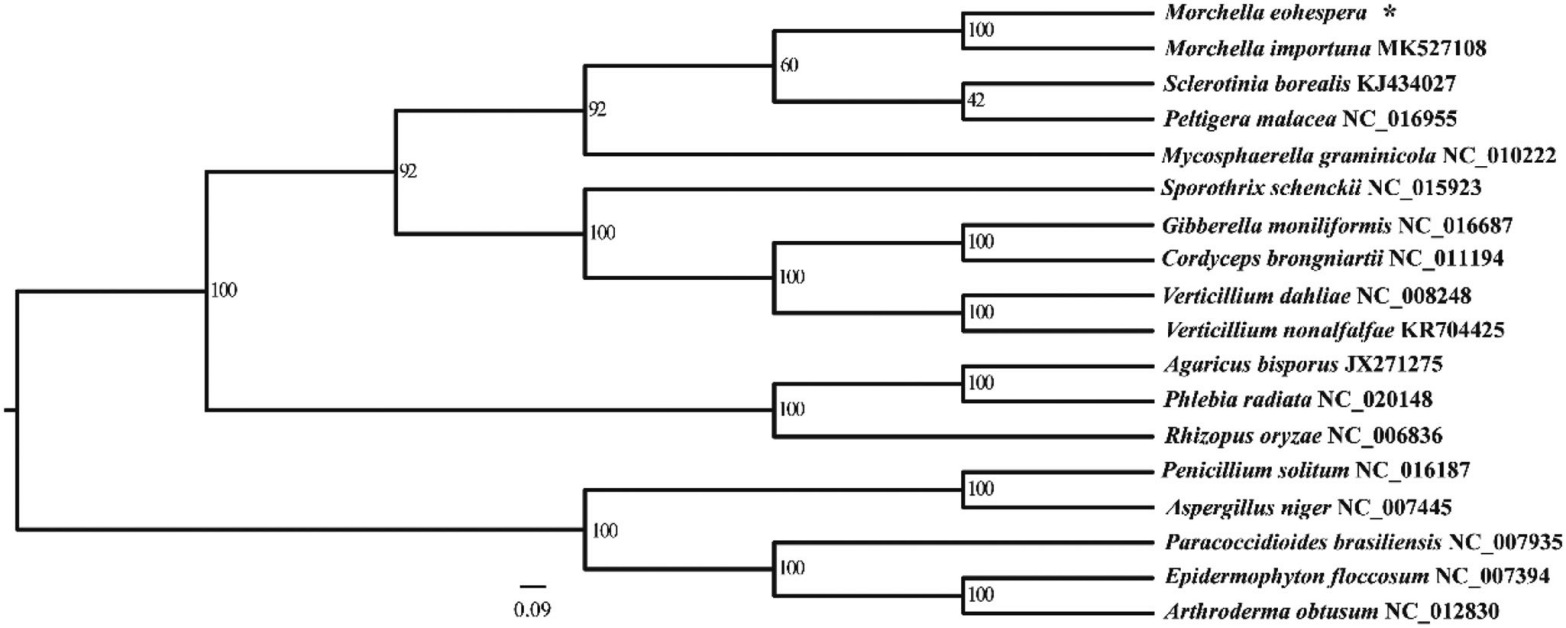
Phylogenetic tree based on fourteen combined mitochondrial protein-coding genes set. *The newly generated mitochondrial genome of *Morchella eohespera*.

## Data Availability

The data that support the results of this research are openly available in GenBank at https://www.ncbi.nlm.nih.gov, reference number MT635617.
